# Overview of Sex and Gender in FTD

**DOI:** 10.1002/alz70857_100826

**Published:** 2025-12-25

**Authors:** Carmela Tartaglia

**Affiliations:** ^1^ Tanz Centre for Research in Neurodegenerative Disease, University of Toronto, Toronto, ON, Canada; Krembil Brain Institute, University Health Network, Toronto, ON, Canada; Toronto Dementia Research Alliance, Toronto, ON, Canada

## Abstract

**Background:**

Across neurodegenerative diseases, sex and gender differences have been increasingly recognized, influencing epidemiology, clinical presentation, biomarker profiles, progression, and response to treatment. Frontotemporal lobar degeneration (FTLD) is the pathological substrate of a heterogeneous group of syndromes characterized by behavioral, language, and motor impairments. Given the heterogeneity of FTLD‐related syndromes, sex and gender differences manifest in distinct ways across its clinical subtypes, including behavioral variant frontotemporal dementia (bvFTD), semantic variant primary progressive aphasia (svPPA), non‐fluent variant PPA (nfvPPA), progressive supranuclear palsy (PSP), corticobasal syndrome (CBS), and frontotemporal dementia (FTD) with motor neuron disease (MND).

**Method:**

This session provides a **comprehensive overview of the influence of sex and gender on FTLD**, integrating insights from epidemiology, genetics, neuroimaging, and biomarker studies

**Result:**

The most consistent sex difference in prevalence is seen in bvFTD, which exhibits a male‐to‐female (M:F) ratio of 2:1 in sporadic cases. However, this sex difference disappears in genetic forms of bvFTD, where prevalence approaches a 1:1 ratio. Similarly, PSP has a male predominance 1.5M:1F. In contrast, sex differences in prevalence are less pronounced in the other FTLD syndromes. Some studies suggest that nfvPPA may be slightly more common in women, but overall, svPPA, CBS, and FTD‐MND do not show consistent sex biases in incidence.

Beyond prevalence, sex‐related differences in clinical phenotype, biomarker profiles, neuroimaging patterns, and disease progression have been reported across FTLD syndromes. Men with bvFTD exhibit more prominent disinhibition, while women often present with more apathy and depression. In PSP, men may experience earlier disease onset, more severe axial rigidity, and faster motor decline, whereas women may have a greater burden of cognitive impairment and bulbar dysfunction. Sex differences in FTLD also depend on genetic status, with specific variations observed across GRN, C9orf72, and MAPT mutations.

Sex differences in CSF and imaging biomarkers, including tau burden, atrophy patterns, and neuroinflammatory markers, are beginning to emerge, highlighting the need for sex‐stratified approaches in FTLD research.

**Conclusion:**

A deeper understanding of sex differences in FTLD may enhance **precision medicine approaches**, inform the development of **more targeted therapies**, and ultimately improve disease management and outcomes for both men and women.

